# Blue light exposure collapses the inner blood-retinal barrier by accelerating endothelial CLDN5 degradation through the disturbance of GNAZ and the activation of ADAM17

**DOI:** 10.1186/s12987-023-00430-7

**Published:** 2023-04-24

**Authors:** Yen-Ju Chan, George Hsiao, Wang-Nok Wan, Tsung-Min Yang, Chi-Hao Tsai, Jaw-Jou Kang, Yu-Cheng Lee, Te-Chao Fang, Yu-Wen Cheng, Ching-Hao Li

**Affiliations:** 1 Department of Physiology, School of Medicine, College of Medicine, Taipei Medical University, 250 Wuxing Street, Taipei, 110 Taiwan; 2 School of Pharmacy, Taipei Medical University, 250 Wuxing Street, Taipei, 110 Taiwan; 3 Department of Pharmacology, School of Medicine, College of Medicine, Taipei Medical University, Taipei, Taiwan; 4 Graduate Institute of Medical Sciences, College of Medicine, Taipei Medical University, Taipei, Taiwan; 5 TMU Neuroscience Research Center, Taipei Medical University, Taipei, Taiwan; 6 Department of Ophthalmology, School of Medicine, The University of North Carolina at Chapel Hill, Chapel Hill, NC 27599 USA; 7 Institute of Food Safety and Health Risk Assessment, School of Pharmaceutical Sciences, National Yang Ming Chiao Tung University, Taipei, 112 Taiwan; 8 TMU Research Center of Urology and Kidney, Taipei Medical University, Taipei, Taiwan; 9 Division of Nephrology, Department of Internal Medicine, School of Medicine, College of Medicine, Taipei Medical University, Taipei, Taiwan; 10 Division of Nephrology, Department of Internal Medicine, Taipei Medical University Hospital, Taipei Medical University, Taipei, Taiwan

**Keywords:** Blue light, Retinal endothelial cells, CLDN5, Inner blood-retinal barrier, GNAZ, ADAM17

## Abstract

Blue light is part of the natural light spectrum that emits high energy. Currently, people are frequently exposed to blue light from 3C devices, resulting in a growing incidence of retinopathy. The retinal vasculature is complex, and retinal vessels not only serve the metabolic needs of the retinal sublayers, but also maintain electrolyte homeostasis by forming the inner blood-retinal barrier (iBRB). The iBRB, which is primarily composed of endothelial cells, has well-developed tight junctions. However, with exposure to blue light, the risks of targeting retinal endothelial cells are currently unknown. We found that endothelial claudin-5 (CLDN5) was rapidly degraded under blue light, coinciding with the activation of a disintegrin and metalloprotease 17 (ADAM17), even at non-cytotoxic lighting. An apparently broken tight junction and a permeable paracellular cleft were observed. Mice exposed to blue light displayed iBRB leakage, conferring attenuation of the electroretinogram b-wave and oscillatory potentials. Both pharmacological and genetic inhibition of ADAM17 remarkably alleviated CLDN5 degradation induced by blue light. Under untreated condition, ADAM17 is sequestered by GNAZ (a circadian-responsive, retina-enriched inhibitory G protein), whereas ADAM17 escapes from GNAZ by blue light illuminance. GNAZ knockdown led to ADAM17 hyperactivation, CLDN5 downregulation, and paracellular permeability in vitro, and retinal damage mimicked blue light exposure in vivo. These data demonstrate that blue light exposure might impair the iBRB by accelerating CLDN5 degradation through the disturbance of the GNAZ-ADAM17 axis.

**Supplementary Information:**

The online version contains supplementary material available at 10.1186/s12987-023-00430-7.

## Introduction

Blue light has wavelengths of 400–500 nm and is known to emit higher energy than other rays in the visible light spectrum. Excessive exposure to blue light is considered hazardous. There is a rapid rise in the use of artificial blue light sources, including 3C displays, light-emitting diode (LED)-based decorative bulbs, and solid-state lighting in our daily lives. The core technology used to produce white light involves pairing a blue LED with a lower-energy phosphor, thereby producing a high proportion of blue light emitted from these sources [1]. Digital devices are prevalent among people across age groups, particularly among adolescents and young adults. The average time spent on mobile devices has been increasing annually. These statistics correlate with the incidence of various illnesses (such as eyestrain, headaches, and fatigue), of which retinopathy is frequent [1, 2].

The retina, with a complex constituent of cell types and layered processes, is a part of the central nervous system (CNS). The outer nuclear layer (ONL) is packed with photoreceptor cell bodies, whereas the inner nuclear layer (INL) contains four retinal neurons (bipolar, horizontal, amacrine, and Müller cells). Synaptic contacts between retinal neurons are localized in the outer and inner plexiform layer (OPL and IPL). The phototransduction cascade is initiated at the photoreceptor outer segments, where stacking membrane disks are enriched with light-absorbing opsins, G-protein transducins, and phosphodiesterases (PDE). These contents are inactive in the dark, resulting in Na^+^ influx via cGMP-dependent cation channel opening. This, in turn, triggers the release of glutamate from the ribbon synapses. Photons activate the opsin-transducin-PDE pathway and reduce cGMP and Na^+^ currents, ultimately leading to membrane hyperpolarization (recorded as a-wave in an electroretinogram [ERG]). Changes in glutamate release are detected by bipolar mGluR6 receptors. The mGluR6 signal allows membrane depolarization of second-order bipolar and horizontal cells in the retinal OPL (ERG b-wave). Similarly, photon-driven signals are conveyed from bipolar cells to the dendrites of retinal ganglion cells in the IPL, and the action potential travels along the optic nerve to the visual cortex [3, 4]. The delicate coupling reactions between G proteins, ion channels, and electrolytes effectively amplify the phototransduction cascade, even when induced by a single photon. Loss of function or disturbance of these factors not only eliminates the ERG phenotype, but also impairs vision processing [5, 6]. In our previous study, rats with periodic blue light exposure not only had decreased total retinal thickness but also impaired retina-to-neuron transduction [7]. Both reactive oxygen species and lipofuscin accumulate in blue-light-treated retinal pigment epithelium (RPE) [8, 9]. The accumulation of oxidative damage may activate the inflammasome, induce endoplasmic reticulum stress, disturb cytosolic calcium, and prevent autophagic flux, eventually reducing the viability of photoreceptors and RPE cells [10–14]. Bioactive ingredients that significantly attenuate oxidative damage induced by blue light also effectively improve cell viability in vitro and prevent retinal degeneration in vivo [10–15]. A multitude of studies have addressed the causation between blue-light-mediated retinopathy and RPE cell (or photoreceptor) degeneration; however, the effects on endothelial cells are limited in the database.

Endothelial cell lines the lumen surface of blood vessels. The abundance of capillary beds in the eye is unsurprising, owing to the metabolically active and high oxygen demanding retina. Retina is nourished by two independent blood-vessel networks. The inner retina is predominantly furnished with stratifying capillary beds in the IPL and OPL, whereas the outer retina is supplied by sheet-like choroidal vessels [16]. The spatial organization of the retinal vasculature let endothelial cells get inevitably exposed to blue light. Therefore, it is important to understand the role of retinal endothelial cells during blue-light-induced retinopathy, especially the physiological changes that respond to low-illuminance that are not cytotoxic. It is well recognized that the endothelium of the retinal vessels is not fenestrated. Retinal endothelial cells are densely joined with each other by forming tight junctions (TJs). With the support from pericytes, Müller cells, and astrocytes, specialized architectures called the inner blood-retinal barrier (iBRB) are formed to maintain the fluid electrolyte balance in the IPL and OPL. iBRB malfunction might cause macular edema, retinal hemorrhage, microaneurysm, and exudation, as seen in various posterior ocular diseases [16, 17]. In diabetic patients, iBRB leakage induces fluid accumulation in the retinal sublayers, consequently disturbing the performance of the ERG, eventually leading to vision impairment. CLDN5 (claudin-5) is an endothelial cell-specific integral component of TJs [18]. In humans, CLDN5 expression fluctuates with neocortical histogenesis. In the early stages, CLDN5 diffuses into the endothelial cytosol and moves to the intercellular junction site by 14–18 weeks of gestation. Subsequently, through interactions between endothelial cells, pericytes, and astrocytes, CLDN5 is expressed continuously and seals paracellularly for barriergenesis [19]. CLDN5 overexpression increases barrier properties in vitro. In contrast, CLDN5 downregulation allows for the rapid permeation of small molecules in vitro and in vivo [20, 21]. In this study, for the first time, we demonstrated that the endothelial CLDN5 protein rapidly degraded upon blue light irradiation; consequently, paracellular permeability increased in vitro. Mice receiving blue light exposure also displayed alleviated ERG b-wave and oscillatory potentials (OPs), attributed to iBRB leakage in vivo. We also explored the G protein subunit alpha Z (GNAZ) to a disintegrin and metalloprotease 17 (ADAM17) axis. Blue light releases ADAM17 from GNAZ, thereby contributing to ADAM17-dependent CLDN5 degradation.

## Materials and methods

### Cell culture, chemicals, and blue light exposure

bEnd.3 were purchased from the Bioresource Collection and Research Center (BCRC, Hsinchu, Taiwan) and maintained in medium formulation suggested by the American Type Culture Collection (ATCC, Manassas, VA). Primary human retinal microvascular endothelial cells (HREC) and endothelial growth medium were obtained from CellBiologics (Chicago, IL, USA). HREC were grown on gelatin-coated dishes. In the routine protocol, cell culture medium was refreshed every 2 days, and cells were passaged at 80–90% confluence. The main findings of this study were authenticated by both bEnd.3 and HREC. The knockdown of ADAM17 and GNAZ was achieved by transient transfection of the pLKO.1 vector with shRNA target sequences (RNA Technology Platform and Gene Manipulation Core, Academia Sinica, Taiwan). Stable clones were produced using puromycin selection. The TAPI-2 (ADAM17 inhibitor) was acquired from Cayman Chemical Company (Ann Arbor, MI, USA), and the stock was prepared in DMSO. The design of the blue light exposure chamber for in vitro studies is shown in Additional file 1: Fig. S1A. Briefly, blue LEDs were oriented at the bottom of the chamber, and the illuminances (80, 160, and 240 lx) were controlled by setting the distance to the LED-emitting plate. The illuminances used in this study are common among modern 3C displayers (Additional file 1: Fig. S1B).

### Cell viability determination (MTT assay and crystal violet staining)

The percentage of live cells was determined by measuring succinate dehydrogenase (SDH) activity in the conversion of 3-(4,5-dimethylthiazol-2-yl)-2,5-diphenyltetrazolium bromide (MTT) to formazan crystals [22]. The relative cell density was quantified by crystal violet staining.

### Immunoblotting

Confluent cells were conditioned in a blue light exposure chamber at indicative illuminances or time intervals. Next, the protein expression level was detected by western blotting, using a previously described protocol [22]. The antibodies used in this study were as follows: anti-CLDN5 (Abcam, Cambridge, UK), anti-ADAM17, anti-ACTB (Sigma-Aldrich, Chicago, IL), anti-ADAM17 (phospho T735) (Cusabio, Houston, TX), anti-GNAZ (Genetex, Irvine, CA), anti-OCLN (Proteintech, Chicago, IL), and anti-TJP1 (Invitrogen, Waltham, MA, USA).

### Transmission electron microscopy (TEM)

The samples for TEM were processed in accordance with the standard protocol of the TMU imaging core facility. TEM images were obtained using a Hitachi HT-7700 microscope.

### Transendothelial electrical resistance (TEER) measurement

The endothelial barrier function was measured in vitro using TEER. The basic concept is that when confluent cells are tightly connected, they resist the passage of electrical current, resulting in high ohmic resistance. The endothelial monolayer grown on a Transwell insert (Falcon, catalog No.353059, 0.4 μm pore size) was exposed to blue light 4–5 days after seeding. Ohmic resistance was measured using an EVOM Epithelial Volt/Ohm Meter equipped with an EndOhm chamber (World Precision Instruments, Sarasota, FL, USA) [22]. The normalized TEER values were corrected for the background and expressed as Ω·cm^2^.

### ADAM17 (TACE) activity assay

ADAM17 enzymatic activity was measured using a fluorometric TACE Activity Assay Kit (Biovision, Milpitas, CA, USA). Briefly, whole-cell lysates were prepared in naive RIPA buffer. For each reaction, 20 μg of protein and FRET substrate were mixed well in a commercial assay buffer, in accordance with the manufacturer’s instructions. It is essential to prepare an additional inhibitor control (TAPI-2). Subsequently, the fluorescence of either the testing group or the inhibitor control was recorded kinetically for 60 min at 318/449 nm (Ex/Em). ADAM17 activity was calculated as relative fluorescence units (RFU) of the testing group to subtract the RFU of the inhibitor control.

### Immunofluorescent staining

Cells were grown on glass coverslips and illuminated with blue light. After the routine protocol involving PBS washing, ice-cold methanol fixation, and blocking, the samples were incubated overnight (4 °C) with primary antibodies, which included anti-ADAM17 (1:50 dilution; Santa Cruz, Dallas, TX, catalog no. sc-390859), anti-GNAZ (1:100 dilution; Cusabio, Houston, TX, catalog no. CSB-PA002856), and anti-CLDN5 (1:100 dilution; Invitrogen, Waltham, MA; catalog no. 34–1600). The samples were washed three times and incubated with a secondary antibody (1:1000) at room temperature for 1–2 h in the dark. Before mounting, the samples were incubated with DAPI for a short period of time to visualize the nuclei. Fluorescent images were captured using a Leica TCS SP5 confocal spectral microscope.

### Immunoprecipitation

After the pre-cleaning process, whole-cell lysates (0.5 mg per reaction) were incubated overnight (4 °C) with 1–2 μg of immunocapturing antibody, which included anti-ADAM17 (Bioss Antibodies, Woburn, MA, catalog no. bs-4236R) and anti-GNAZ (Genetex, Irvine, CA, catalog no. GTX11444). The next day, protein G magnetic beads (Merck Millipore, Burlington, MA) were added and incubated for another 2 h. All the incubations were performed on a rotating shaker at a 4 °C refrigerator. Finally, the immunocomplexes were washed three times and separated using SDS-PAGE. Immunoblotting was performed according to the standard procedures.

### Animal husbandry and treatment

The animal handling protocol was approved by the IACUC facility (LAC-2020-0482), and the experimental flowchart is shown in Additional file 1: Fig. S2. C57BL/6 mice (6 weeks old, SPF grade, obtained from BioLASCO, Taipei, Taiwan) were grouped and housed at Taipei Medical University Animal Center. After 1-week acclimation, the mice were subjected to ophthalmology examination battery, including fundus photography (FP), fundus fluorescein angiography (FFA), spectral domain-optical coherence tomography (SD-OCT), and ERG, and denoted as day 1. For the light exposure study, mice were conditioned in a blue (or red) light exposure chamber (6 h per day; from 10 AM to 4 PM) for consecutive 3 days (days 2–4). On day 5, the ophthalmology examination battery was re-evaluated. For GNAZ-knockdown study, mice received a single intravitreal (ITV) injection of 1 μL mixture of GNAZ shRNA (20 ng per eye) and jet^OPTIMUS^ transfection reagent in their right eye. The sham treatment was performed on the left side. An ophthalmology examination battery was conducted 5 days after ITV injection. The mice were then euthanized. Their eyeballs were isolated, and homogenates were prepared as previously described. GNAZ knockdown in vivo was validated by immunoblotting.

### Fundus photography (FP), fundus fluorescein angiography (FFA), and spectral domain-optical coherence tomography (SD-OCT)

FP, SD-OCT, and FFA images were acquired sequentially using the Phoenix Micron III system (Tempe, AZ, USA), according to the manufacturer’s instructions. The mice were anesthetized with a mixture of ketamine and xylazine, followed by a topical administration of 0.125% atropine for pupil dilation. For improved visualization of the fundus, a topical drop of 2% Methocel^®^ (OmniVision, Switzerland) was administered before ophthalmoscopic observation. After SD-OCT scanning, an intraperitoneal injection of sodium fluorescein (10 mg/kg) was administered prior to FFA observation. To quantify structural changes, retinal thickness (or retinal sublayers) was automatically computed using the Insight software, as described previously [22]. The FFA images for the quantification analysis were saved in the 16-pixel format. After the removal of the fluorescence signal in the retinal vessels, the remaining areas (in terms of the number of affected pixels) are considered the leakage regions. The total leakage area was calculated using ImageJ (U.S. National Institutes of Health, Bethesda, MD, USA) [23].

### Scotopic electroretinogram (ERG)

ERGs were performed using the Diagnosys Celeris^™^ ERG system (Diagnosys LLC) and preset programs. A day before ERG testing, the mice were dark-adapted for > 12 h. Animal handling was performed under dim red light. Anesthetized mice were positioned on the thermal pad (37 ± 1 °C), and the stimulator was placed on the surface of the cornea with the largest contact surface, according to the protocol suggested by the instructor [24]. The dark-adapted ERG and OPs were stimulated with an ascending flash series (intensity 0.01, 0.1 and 1 cd.s/m^2^; frequency 1 Hz), and the signal waves were acquired from 50 ms pre-trigger to 300 ms post-trigger. The stimulus parameters of the c-wave ERG were 150 cd.s/m^2^ and 1 Hz, and the signal waves were acquired from 20 ms pre-trigger to 10000 ms post-trigger. The a-, b-, and c-wave amplitudes (µV) and implicit times (ms) were automatically detected using the Espion software (version 6; Diagnosys LLC).

### Statistical analysis

All data are expressed as mean ± standard error of the mean (S.E.M.). Differences between groups were analyzed using the unpaired Student’s *t*-test or one-way analysis of variance (ANOVA), followed by Dunnett’s multiple comparison test. *p < 0.05 and **p < 0.01 or ***p < 0.001 represents statistical significance.

## Results

### The variable cytotoxicity induced by blue light depends on illuminance and exposure time

bEnd.3 cells or HRECs were exposed to blue light (80, 160, and 240 lx) for 6, 12, and 24 h; afterward, cell viability was evaluated by either the cell density in culture (crystal violet assay) or SDH activity (MTT assay). The 240 lx blue light illuminance > 12 h or 24 h exposure to blue light > 160 lx was sufficient to reduce cell density and SDH activity. In addition, 80 lx illuminance for 24 h significantly reduced SDH activity in the treated endothelial cells (Table 1). These conditions are not suitable for in vitro studies on endothelial barrier function. An illuminance of 160 lx for 6–12 h did not reduce the density of the endothelial cells and was considered the predominant testing condition. This illuminance is frequently achievable in 3C displayers and is suitable for a general reading distance (Additional file 1: Figure S1B).

**Table 1:** Blue-light-induced cytotoxicity depends on the illuminance and exposure time

Exposure time (h)	Cell density (counts/field)			SDH activity (% of control)		
	80 lx	160 lx	240 lx	80 lx	160 lx	240 lx
bEnd.3 cell						
0	284.8 ± 6.4			100.2 ± 0.2		
6	288.3 ± 14.3	283.9 ± 10.3	295.6 ± 4.8	97.6 ± 1.3	96.7 ± 1.9	82.8 ± 2.8***
12	288.0 ± 21.2	277.9 ± 9.4	222.3 ± 6.3***	94.5 ± 4.4	84.2 ± 1.1**	69.8 ± 2.4***
24	273.0 ± 9.0	232.8 ± 21.7*	195.4 ± 6.2***	79.8 ± 3.5***	58.5 ± 8.7***	32.2 ± 14.8***
HREC						
0	243.1 ± 10.7			100.0 ± 0.0		
6	236.3 ± 16.6	236.8 ± 9.2	214.9 ± 5.8	98.9 ± 4.8	100.6 ± 3.7	97.8 ± 3.6
12	234.9 ± 14.5	231.5 ± 9.9	195.3 ± 11.5*	100.4 ± 5.5	94.8 ± 3.8	81.9 ± 5.1**
24	223.5 ± 14.1	192.5 ± 8.3*	175.5 ± 14.8**	87.5 ± 3.3**	66.8 ± 7.5**	57.1 ± 12.2**

Cell viability was determined by cell density (crystal violet assay) and succinate dehydrogenase (SDH) activity (MTT assay)

Blue light reduced SDH activity and cell density at 240 lx exposure for > 6 h (bEnd.3) and > 12 h (HREC). In addition, 24 h blue light exposure > 80 lx significantly reduced SDH activity in both endothelial cell lines. Therefore, exposure to 240 lx illuminance and a 24 h exposure period were not favorable for further experiments. The endothelial cells were less viable but were still alive after exposure to 160 lx blue light for 12 h. The conclusions drawn from the in vitro data were mostly dependent on the 160 lx. This illuminance is frequently achievable in 3C devices and is feasible in terms of reading distance

^*^p < 0.05, **p < 0.01, ***p < 0.001, indicates statistical difference from the control

### Blue light reduces endothelial CLDN5 protein level and deteriorates paracellular TJ integrity

**Fig. 1:**
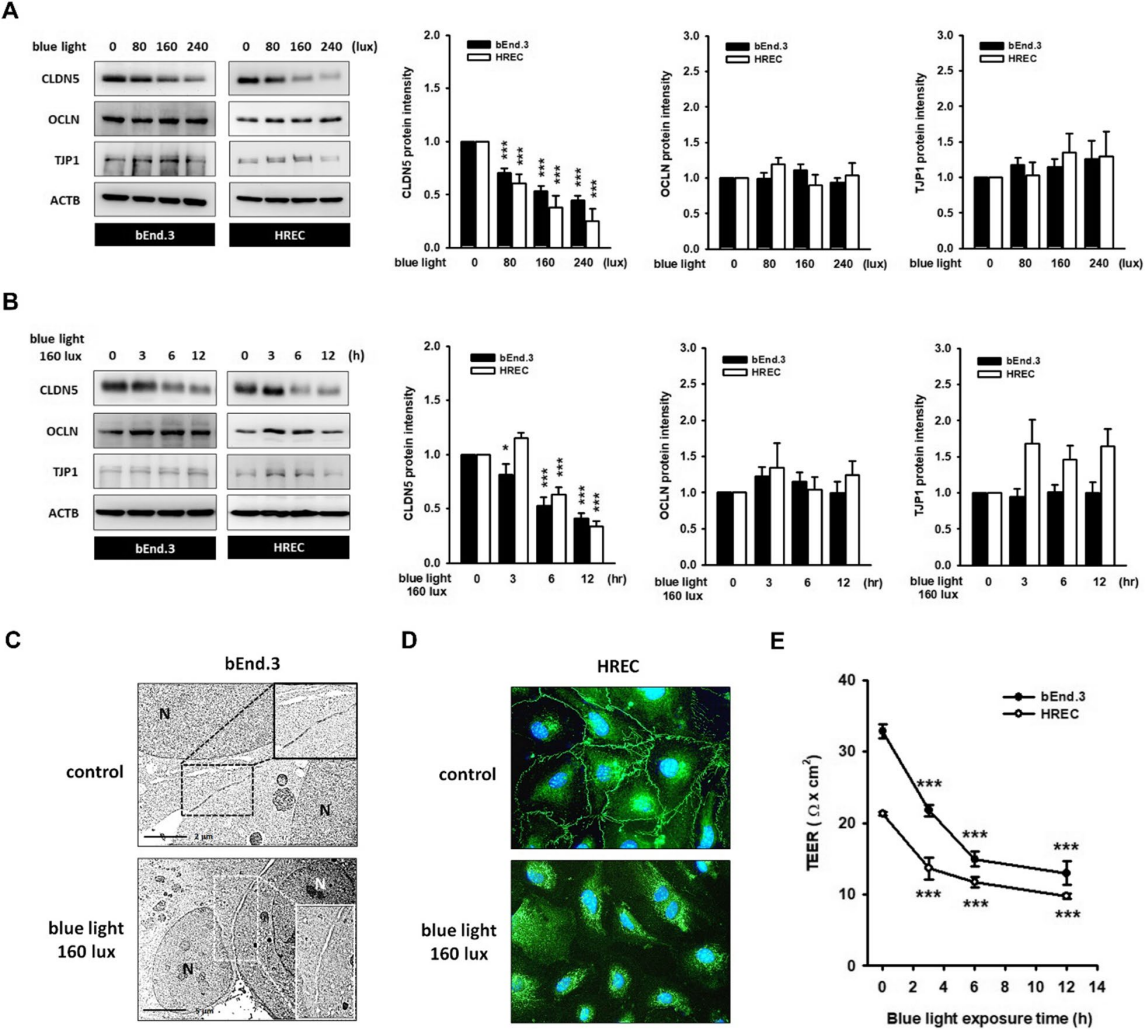
Blue light reduces CLDN5 protein level, destroys tight junction (TJ), and impairs the physiology barrier function of endothelial cells. **A** The bEnd.3 and HREC monolayer were exposed to blue light (80, 160, and 240 lx) for 12 h, followed by routine immunoblotting. Representative images and histograms showed an obvious CLDN5 protein downregulation in illuminance-dependent manner. **B** CLDN5 protein began to degrade upon 3–6 h, 160 lx blue light exposure. The expression level of TJP1 and OCLN remained unaltered. (*p < 0.05, ***p < 0.001, indicates statistical difference from the control treatment). **C** TEM images showed a clear TJ ultrastructure in the border of adjacent bEnd.3 cells. After 12 h and 160 lx blue light exposure, the TJ almost disappeared, and an obvious cleft between adjacent cells was observed. The N represents the nucleus. **D** The CLDN5-positive strands that appeared at the border of HREC membrane became discontinuous under blue light, suggesting that TJs were damaged. **E** TEER values were measured as described in Materials and Methods to evaluate the barrier function. TEER value across the bEnd.3 cell and HREC monolayer was notably reduced with time after 160 lx blue light exposure (***p < 0.001, indicates statistical difference from the control). These data supported the loss of the physiology barrier in blue-light-treated endothelial cells

Endothelial TJs serve as important physiological barriers that restrict the movement of water and small molecules through the paracellular cleft. Upon blue light exposure, we found that CLDN5 protein, the key constituent of endothelial TJ, was downregulated in an illuminance-dependent and time-dependent manner (Fig. 1A, B). Following a 6 h, 160 lx blue light irradiation, the CLDN5 protein level was reduced to 0.53 ± 0.08 (bEnd.3) and 0.63 ± 0.07 (HREC), compared with that of the controls. As shown in Fig. 1C, TEM images showed an apparent TJ ultrastructure between neighboring bEnd.3 cells, whereas the TJs disappeared, and a gap appeared instead upon blue light exposure. CLDN5 was present at the HREC membrane border of the control, and its appearance became discontinuous under blue light (Fig. 1D). Both sets of data support a broken structure in the TJ plaques. In vitro paracellular permeability was evaluated by measuring the TEER. A significant reduction in the TEER value was initially found after 3–6 h blue light exposure (Fig. 1E), which aligned with the CLDN5 downregulation. The loss of barrier function in blue-light-treated endothelial cells was also confirmed by the diffusion of FITC-dextrin (data not shown). These data demonstrated that blue light exposure caused rapid CLDN5 downregulation, conferring to the deterioration of endothelial TJs and their barrier function.

### Blue light activates ADAM17 by increasing T735 phosphorylation

**Fig. 2:**
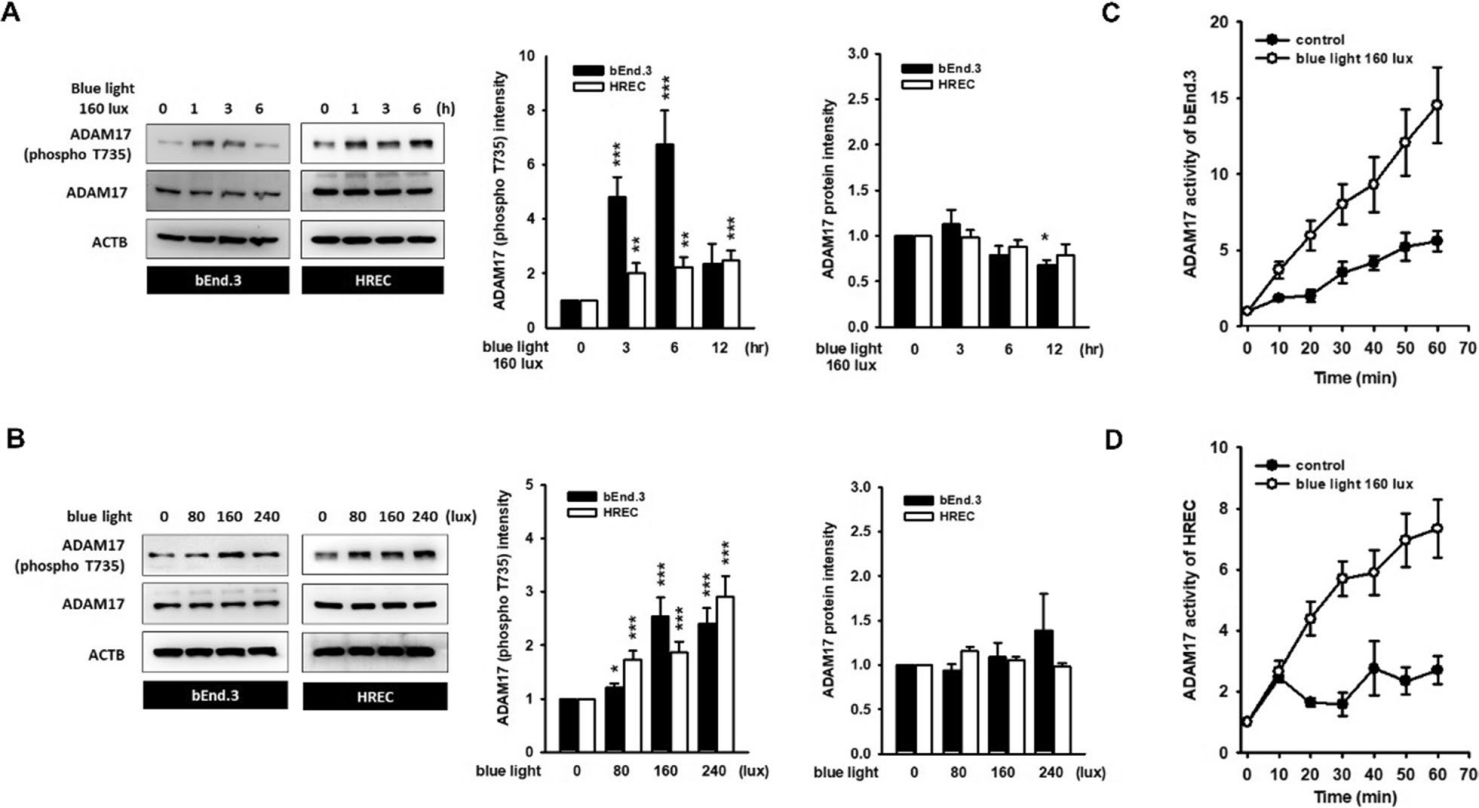
Blue light induces ADAM17 phosphorylation and activates ADAM17 in treated endothelial cells. After blue light stimulation, the phosphorylation level of ADAM17 on T735 was detected by immunoblotting. Representative images and histograms showed a remarkable phosphorylation of ADAM17 in **A** illuminance-dependent and **B** time-dependent manner. (**p < 0.01, ***p < 0.001, indicates statistical difference from the control group). **C**, **D** ADAM17 kinetics assay was performed by fluorometric method in accordance with the manufacture’s protocol. The kinetic curve of resting endothelial cells was flattened with incubation time, whereas the steep slope of group with blue light (160 lx, 1 h) stimulation represents the rapid activation of ADAM17 by blue light. **C** bEnd.3 cell. **D** HREC

Phosphorylation of the T735 residue in ADAM17 cytoplasmic domain is a crucial step in its shedding activity [25, 26]. We found that ADAM17 starts to phosphorylate at the T735 residue after 1 h of 160 lx blue light stimulation, whereas phosphorylation peaked at 3 h (bEnd.3) or 3–6 h (HREC) of exposure (Fig. 2A). Blue-light-induced ADAM17 phosphorylation was confirmed in illuminance-dependent manner (Fig. 2B). The kinetics of ADAM17 activity measured by the fluorogenic substrate showed a slope in the blue-light-treated groups, as compared to the flattened curve of the control treatment (Fig. 2C, D). These data demonstrate that blue light rapidly activates ADAM17. Moreover, neither the degradation of CLDN5 nor the phosphorylation of ADAM17 occurred in endothelial cells treated with red light (Additional file 1: Fig. S3), suggesting that the responses are specific to blue light.

### Blue-light-mediated CLDN5 degradation is dependent on ADAM17 activity

**Fig. 3:**
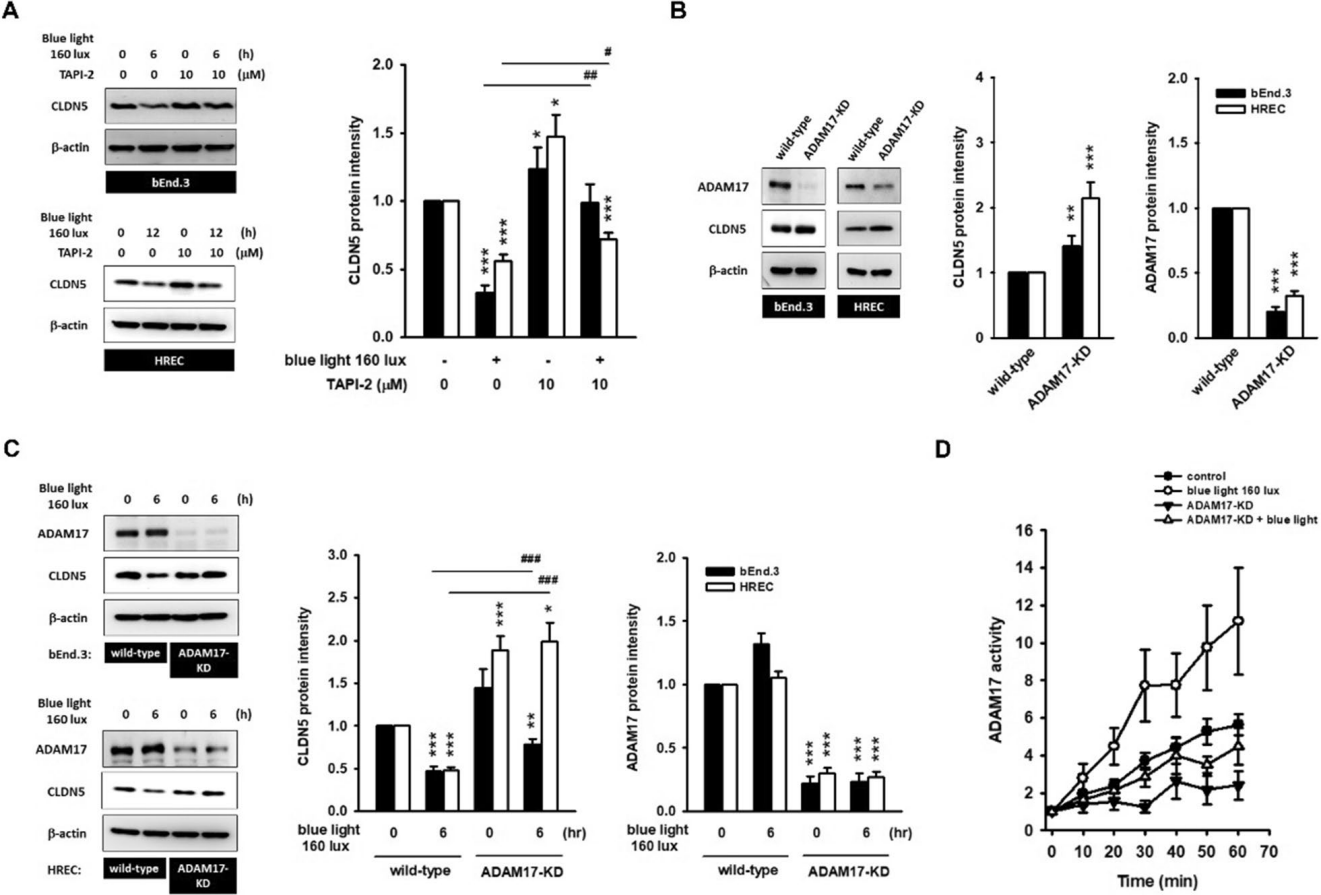
Blue-light-mediated CLDN5 degradation is rely on ADAM17 activity. **A** Blue-light-mediated CLDN5 degradation could be prevented by 2 h pre-incubation of TAPI-2 (ADAM17 inhibitor, 10 μM). (*p < 0.05, ***p < 0.001, indicates statistical difference from the control treatment; ^#^ < 0.05, ^##^p < 0.01 indicates statistical difference from the blue-light-treated group). **B** bEnd.3 ADAM17-KD (stable clone) and HREC ADAM17-KD (transient transfection) were built. The knockdown of ADAM17 was accompanied by the CLDN5 protein accumulation, suggesting the involvement of ADAM17 activity in regular CLDN5 turnover. **C** Blue-light-mediated CLDN5 degradation was completely prevented in either bEnd.3 ADAM17-KD, or in HREC ADAM17-KD, as compared to that in wild-type. (*p < 0.05, **p < 0.01, ***p < 0.001, indicates statistical difference from the wild-type control treatment; ^###^p < 0.001 represents the significant difference from the blue-light-treated group). **D** In bEnd.3 ADAM17-KD clones, the enzymatic kinetics of ADAM17 was not induced in respond to blue light exposure. These data provide substantial evidence demonstrating the blue-light-mediated endothelial CLDN5 degradation, involving ADAM17 activation

To determine the effect of ADAM17 on CLDN5 degradation, endothelial cells were pre-incubated with TAPI-2 (10 μM; ADAM17 inhibitor) for 2 h, followed by exposure to 160 lx blue light for 6 h. Pronounced CLDN5 degradation was observed in blue-light-treated group, but CLDN5 protein levels were retained by adding TAPI-2 (Fig. 3A). Next, blue-light-mediated CLDN5 degradation was re-evaluated using ADAM17-knockdown (ADAM17-KD) systems. As shown in Fig. 3B, ADAM17-KD was successfully built into bEnd.3 (stable) and HREC (transient). In both testing systems, ADAM17 silencing was accompanied by apparent CLDN5 upregulation. This indicates a regular CLDN5 turnover process by ADAM17. Subsequently, the CLDN5 protein level was notably retained in ADAM17-KD systems, even after exposure to blue light (Fig. 3C). As predicted, the blue-light-mediated ADAM17 kinetics were diminished in the ADAM17-KD system (Fig. 3D).

Both ADAM9 and ADAM10 are expressed in retinal endothelium [27, 28]. However, the knockdown of ADAM9 did not rescue blue-light-mediated CLDN5 degradation. ADAM10 was dramatically decreased by blue light; furthermore, ADAM10 knockdown enhanced CLDN5 degradation in combination with blue light exposure (Additional file 1: Fig. S4). Thus, we hypothesized that ADAM17 is a unique, blue-light-responsive player in endothelial cells that regulates barrier function by processing CLDN5 turnover.

### Blue light dissociates the GNAZ-ADAM17 interaction, leading to ADAM17 activation and CLDN5 degradation

**Fig. 4:**
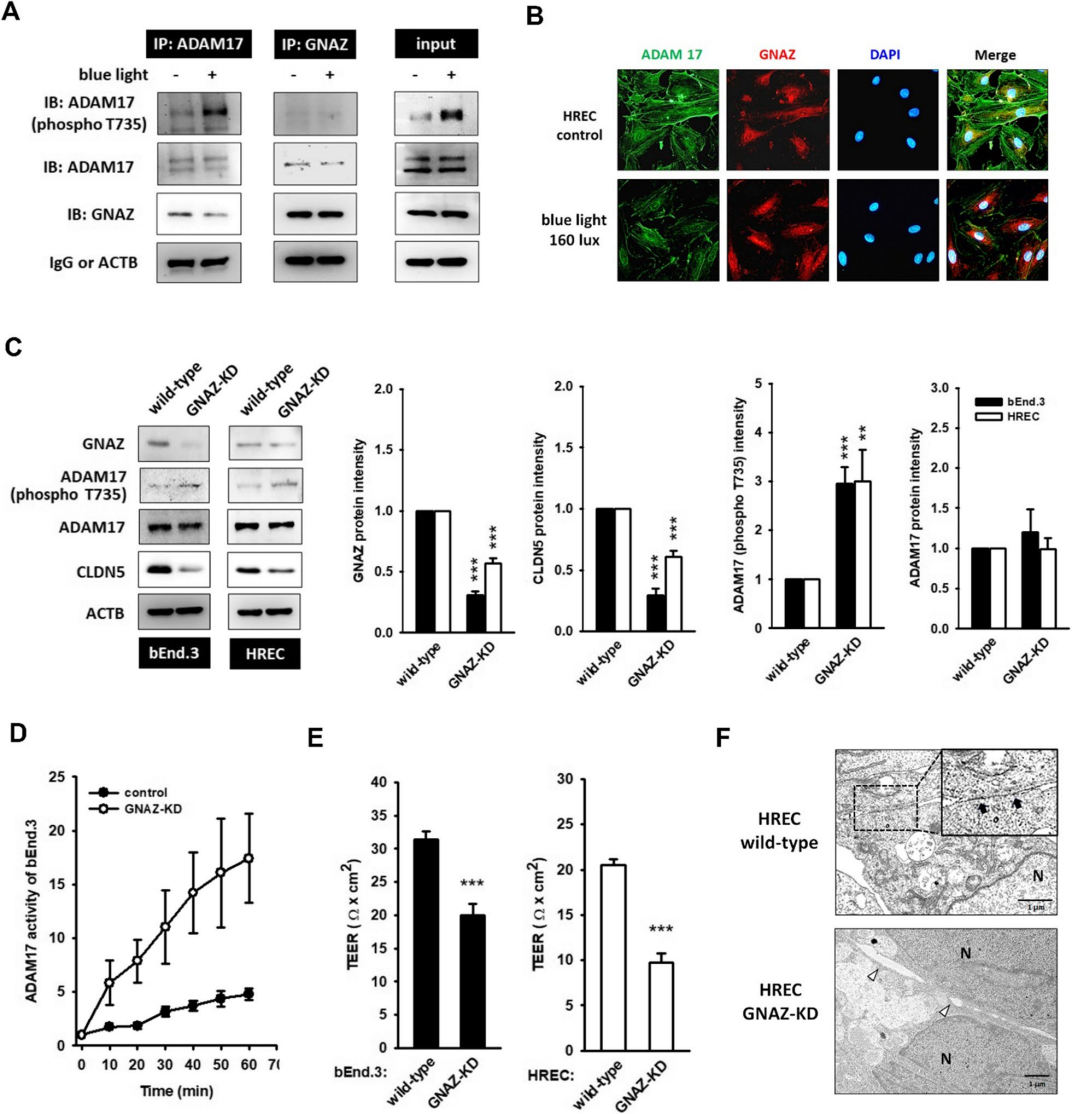
GNAZ is a negative regulator of ADAM17. G protein subunit alpha Z (GNAZ), which is ubiquitously expressed in retinas, is thought to be regulated by the biological clock. **A** The co-immunoprecipitation data demonstrated the association between GNAZ and ADAM17 in untreated bEnd.3 cell. However, blue light exposure (160 lx, 3 h) seemed to disturb the protein–protein interaction. In addition, the GNAZ/ADAM17 protein complex is harmful for ADAM17 phosphorylation. **B** Immunofluorescent staining images showed the co-localization of GNAZ (red) and ADAM17 (green) on the cell membrane of untreated HREC. After blue light stimulation (160 lx, 3 h), GNAZ moved from the cell membrane to the cytosol. The nucleus was stained by DAPI (blue). **C** Silencing GNAZ was achieved by the transfection of shRNA. The successful knockdown of GNAZ coincided with the downregulation of CLDN5 and the hyperactivation of ADAM17. (**p < 0.01, ***p < 0.001, indicates statistical difference from the control). **D** GNAZ-KD clones were increased in their ADAM17 activity. (E) The TEER value also dropped in GNAZ-KD clones. (***p < 0.001, indicates statistical difference from the wild-type control). **F** TEM images showed an obvious cleft between adjacent HRECs, instead of the appearance of a TJ

The expression of GNAZ, an inhibitory G protein, has been profiled in retinal tissue across species [29–31]; however, their functions have not yet been fully established. It is intriguing that, by using co-immunoprecipitation, the interaction of GNAZ-ADAM17 was identified in resting bEnd.3 cell, whereas blue light exposure (160 lx, 3 h) dissociated the GNAZ-ADAM17 complex, thereby improving ADAM17 phosphorylation levels (Fig. 4A). Next, the colocalization of GNAZ and ADAM17 was observed on the cell membrane of untreated HREC. However, membrane-bound GNAZ dispersed into the cytosol after blue light stimulation (Fig. 4B). These data suggest that GNAZ might be a negative regulator of ADAM17.

To validate this hypothesis, we constructed a GNAZ-KD system. We found that GNAZ knockdown coincided with ADAM17 hyperphosphorylation and CLDN5 downregulation (Fig. 4C). Furthermore, GNAZ-KD systems displayed enhanced ADAM17 activity (Fig. 4D), reduced TEER value (Fig. 4E), and an apparent gap occupying the TJ site (Fig. 4F). Thus, we considered that the activity of ADAM17 was sequestered by GNAZ in resting condition, but exposure to blue light might allow ADAM17 to escape from GNAZ, resulting in the activation of ADAM17 and ADAM17-mediated CLDN5 degradation.

### *Blue light destroys the iBRB and impairs retinal electrophysiology in vivo*

**Fig. 5:**
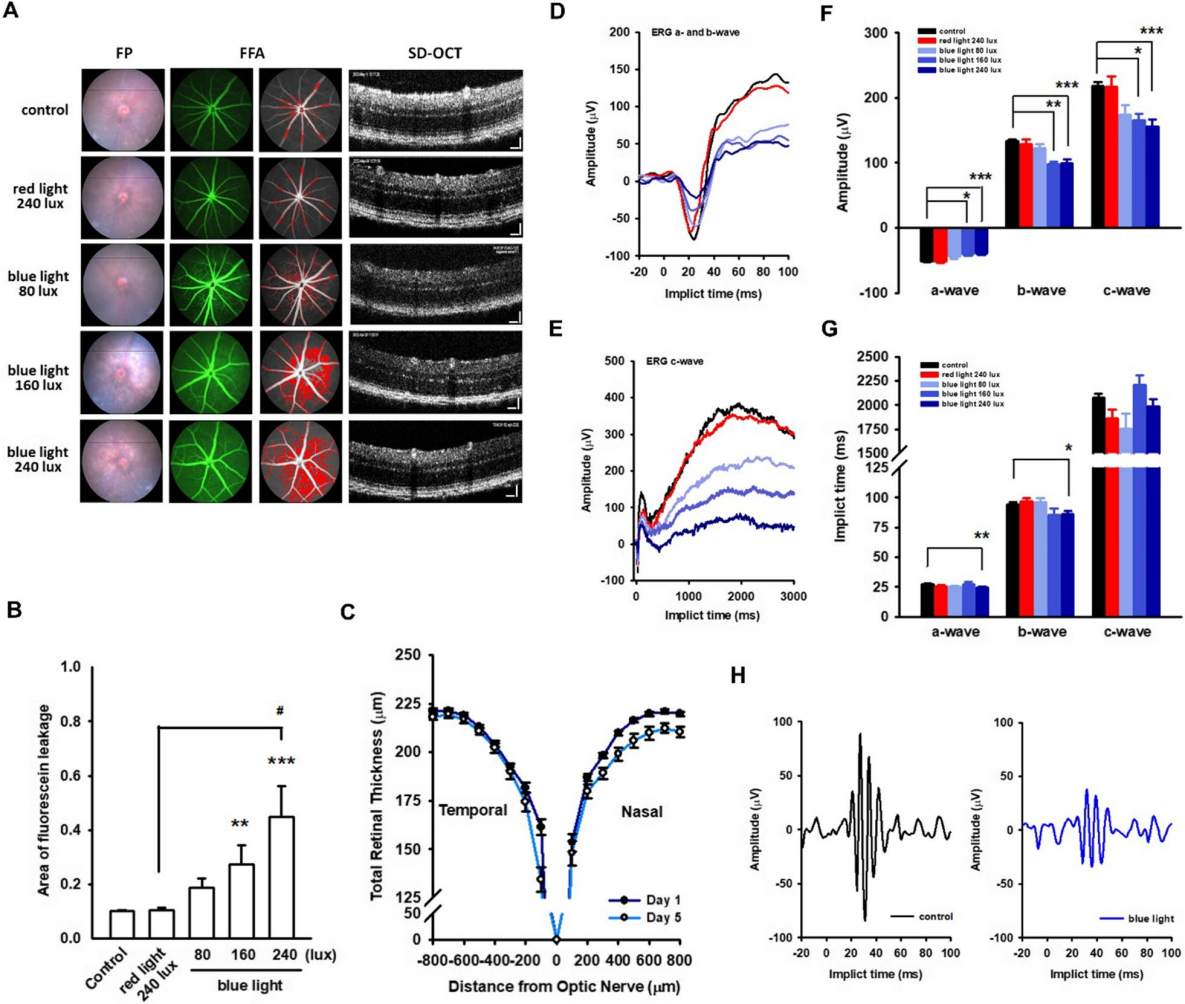
Blue light destroys the iBRB and impairs the retinal electrophysiology in vivo. To observe the retinal injuries in vivo, C57BL/6 mice were exposed to blue light (6 h per day, for consecutive 3 days), and ophthalmology examination battery was performed on days 1 and 5 (Additional file 1: Fig. S2A). **A** The abnormality in fundus and the leaking fluorescein were presented in blue light groups, as compared to the control and red light groups. The area of fluorescein leakage was quantified as described in Materials and Methods. An illuminance-dependent iBRB leakage presented by blue light **B**. Although no obvious damages in retinal architecture were identified in OCT-scanning images, but the retinal thickness seemed to atrophy **C**. The incidence of retinal abnormalities and the mean thickness of retina sublayers were summarized in Additional file 1: Tables S1 and 2, respectively. **D**, **E** Scotopic ERG recordings showed an illuminance-dependent damages in their amplitude after blue light treatments, as compared to either untreated or red-light-treated groups. **F** The quantified data affirmed the attenuation of the ERG amplitude in a-, b-, and c-wave upon blue light irradiation, of which the b-wave is preferentially affected. **G** A delayed ERG implicit time was also identified in 240 lx blue-light-treated group. (*p < 0.05, **p < 0.01, ***p < 0.001, indicates statistical difference from the right eye of control mice). (H) The OPs appearing in the ascending limb of b-wave are thought to reflect the signal transmissions of the inner retina. The amplitude of OPs was dramatically diminished in respond to blue light stimulation. Taken together, blue light exposure impaired the retinal electrophysiology property by disturbing iBRB function

To observe retinal function in vivo, C57BL/6 mice were restrained shortly in a chamber equipped with blue LEDs (80, 160, and 240 lx, exposure for 6 h per day, from day 2 to day 4), and retinal function battery was performed on day 1 (before exposure) and day 5 (post exposure) (Additional file 1: Fig. S2A). Neither histopathological abnormalities nor terminal deoxynucleotidyl transferase dUTP nick end labeling (TUNEL)-related cell death was observed in the retinal sections of any of the test animals (Additional file 1: Fig. S5A). However, the retinas of blue-light-treated groups showed acute functional damages, and the incidence is summarized in Additional file 1: Table S1. Using FFA, leaking fluorescein was observed in the fundus field (incidence: 4/10, 4/10, and 7/10 in the 80, 160 and 240 lx blue-light-treated groups, respectively). The quantified data showed an illuminance-dependent increase in the “area of fluorescein leakage”, thus reflecting the iBRB injuries induced by blue light rather than by red light (Fig. 5A middle panel and 5 B). Retinal architecture destruction was scanned and imaged by SD-OCT with an incidence of 6/10 in the 240 lx blue-light-treated group (Fig. 5A, right panel). The thickness of the total retina (or retinal sublayers) was computed automatically (Table 2). The absolute thickness of total retina was computed from 227.2 ± 1.1 μm (control) to 215.0 ± 5.8 μm (240 lx blue-light-treated, p < 0.001), whereas the retinal thickness was 223.1 ± 5.6 μm in red light (240 lx)-treated group (Fig. 5C and Table 2). To compromise the retinal thickness deviation among individuals, the data also expressed as “% of total retina”. It is clear that atrophy predominantly occurs in the IPL-to-INL and OPL-to-ONL regions, where iBRB is abundant for maintaining fluid homeostasis. Phototransduction performance was recorded using ERG. We found that ERG, either the amplitude or the implicit time, was quite sensitive to blue light irradiation (incidence: 5/10, 4/10, and 8/10 in the 80, 160, and 240 lx blue-light-treated groups, respectively). The amplitude of ERG a-, b-, and c-waves attenuated dramatically in response to blue light exposure, of which the b-wave was the most affected (Fig. 5D–F). In addition, the implicit time was significantly delayed (Fig. 5G). More seriously, the OPs were notably alleviated by exposure to blue light (240 lx) (Fig. 5H). These abnormalities (mild to moderate) were also found in the 80 and 160 lx blue-light-treated groups. Depending on the hazard sensitivity in the b-wave and OPs, which reflect the signal processes in the IPL-to-ONL region, we hypothesized that the disturbance of retinal circulation might be the major cause. This hypothesis is also supported by the loss of CLDN5 and active ADAM17 in the retinal sections of the blue-light-treated groups (Additional file 1: Fig. S5B), as well as the increased area of fluorescein leakage in the fundus.

**Table 2:** Retinal thickness among testing mice

	Treatment				
	Control	Blue light ^1^			Red light 240 lx
		80 lx	160 lx	240 lx	
Total retinal thickness	227.2 ± 1.1	216.6 ± 2.9***	215.0 ± 6.6**	215.0 ± 5.8***	223.1 ± 5.6
Retinal sub-layers analysis					
IPL-to-INL					
mean thickness (μm)	97.2 ± 0.4	91.0 ± 3.3***	90.2 ± 4.4**	89.1 ± 3.1***	95.1 ± 3.5
% of total retina	42.8 ± 0.2	41.9 ± 1.2	41.8 ± 1.1	41.4 ± 0.9*	42.6 ± 0.7
OPL-to-ONL					
mean thickness (μm)	73.8 ± 0.4	70.8 ± 2.3	68.7 ± 5.0	64.8 ± 4.0***	76.6 ± 2.0
% of total retina	32.5 ± 0.2	32.6 ± 0.9	31.5 ± 1.6	29.9 ± 1.4***	34.5 ± 1.6
IS/OS					
mean thickness (μm)	43.6 ± 0.6	41.1 ± 2.3	43.1 ± 2.7	45.7 ± 2.4	38.5 ± 3.5*
% of total retina	18.7 ± 0.3	19.0 ± 1.2	20.6 ± 2.1	21.4 ± 1.3**	17.1 ± 1.2
RPE					
mean thickness (μm)	13.3 ± 0.2	13.8 ± 0.8	13.0 ± 0.4	14.2 ± 0.9	12.8 ± 0.6
% of total retina	5.9 ± 0.1	6.4 ± 0.6	6.2 ± 0.4	6.7 ± 0.6*	5.8 ± 0.3

During blue light exposure (80, 160, and 240 lx), C57BL/6 mice were placed in the exposure chamber for 6 h per day (10 AM to 4 PM) for three consecutive days (from day 2 to day 4). An ophthalmology examination battery was performed on days 1 (before exposure) and 5 (post-exposure)

Retinal thickness was computed automatically by the software

Data are expressed as mean ± S.E.M. **p < 0.01, ***p < 0.001, indicates statistical difference from the control group. The N value are listed in Additional file 1: Table S1

### *In vivo GNAZ knockdown induced retinal damage that mimicked blue light exposure*

**Fig. 6:**
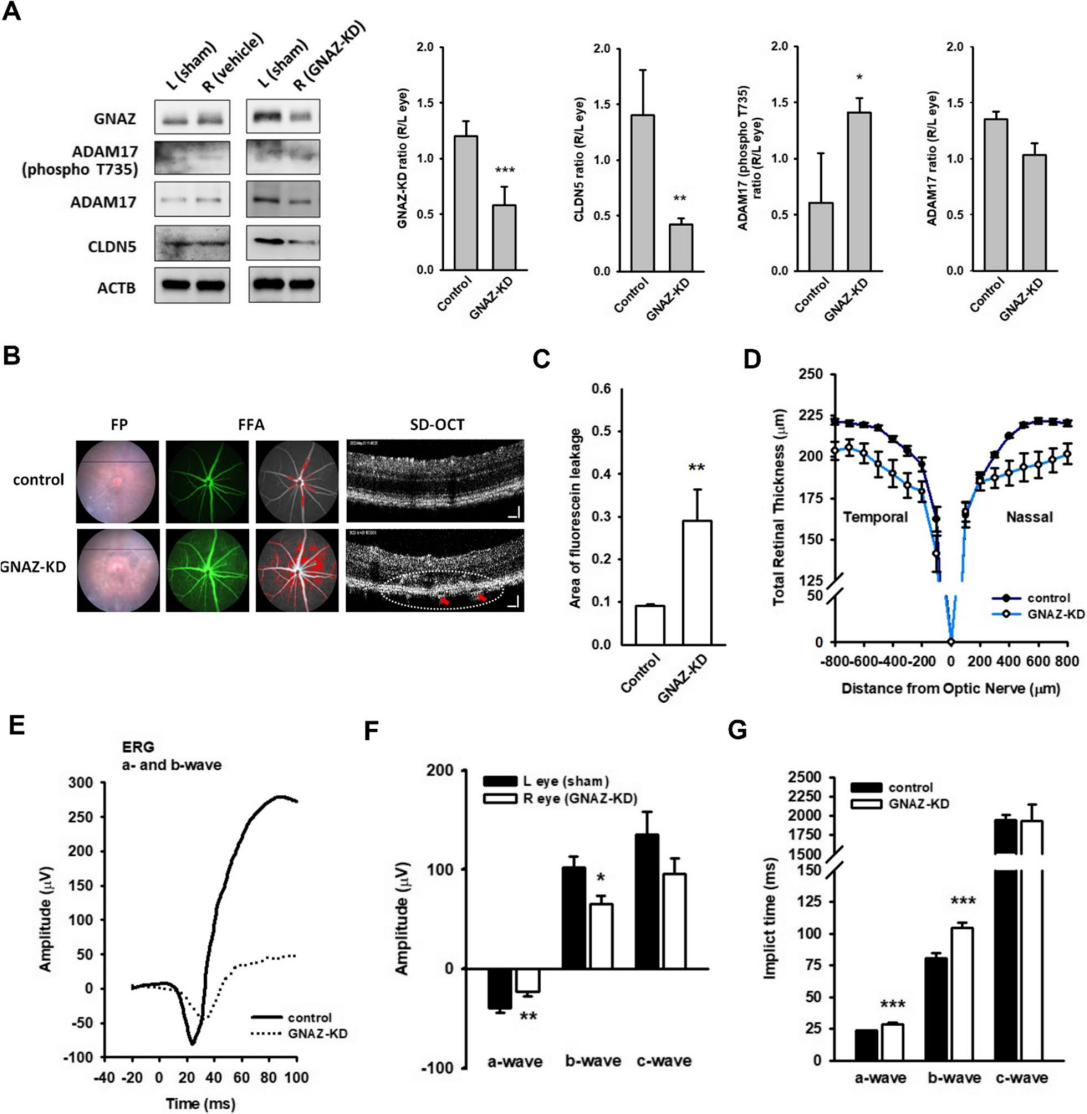
In vivo GNAZ knockdown induces retinal damages that mimic blue light exposure. To study the role of GNAZ, the retinal GNAZ was silenced by ITV injection of shRNA, subjected to ophthalmology examination (Additional file 1: Figure S2B). **A** Representative images and the quantified histogram authenticated the knockdown of GNAZ in the right eye (shRNA-injected), as compared to that in the left eye (sham treatment). The right eye also exhibited an increased phospho-ADAM17 and a reduced CLDN5 protein level. (*p < 0.05, **p < 0.01, ***p < 0.001, indicates statistical difference from the right eye of control mice). **B** Several exudates were observed in FP of GNAZ-KD group. By FFA, the leaking fluorescein were found in the fundus field, corresponding to the quantification data **C**. OCT-scanning proved the exudates in GNAZ-KD group. **D** Retinal thickness computed by the software showed an atrophy of total retina by GNAZ-KD, as compared to that in the control. The thickness of retinal sublayers was summarized in Table 3. **E** In GNAZ-KD, the recording ERG a- and b-wave were obviously attenuated in amplitude **F** and delayed in implicit time **G**. (*p < 0.05, **p < 0.01, ***p < 0.001, indicates statistical difference from the sham eye). These data strongly indicate that GNAZ might be the gatekeeper of retinal electrophysiology function in vivo. GNAZ-KD induced retinopathy mimic blue light exposure

Retina-specific gene silencing can be achieved using ITV shRNA delivery [32]. Additional file 1: Fig. S2B shows a flowchart of the animal treatments. We found reduced GNAZ levels in the right eye, accompanied by ADAM17 hyperphosphorylation and CLDN5 downregulation, compared to the left eye (sham treatment) (Fig. 6A). Four of the six GNAZ-KD mice showed exudates in the fundus field, which were aligned with an obvious leakage of fluorescein (Fig. 6B, left and middle panels; Additional file 1: Table S1). The area of fluorescein leakage was significantly increased by GNAZ-KD, as compared to that in the control group (Fig. 6C). OCT-scanning data showed the severe retina atrophy with the mean thickness of 231.3 ± 0.9 μm (control) and 218.4 ± 1.7 μm (GNAZ-KD, p < 0.001), respectively (Fig. 6D and Table 3). The IPL-to-INL appeared to swell, whereas the IS/OS region was squeezed. Finally, the recording ERG performance was dampened, particularly the b-waves (Fig. 6E–G). These data strongly indicate that GNAZ may be the gatekeeper of retinal electrophysiological function in vivo.

**Table 3:** Retinal thickness in control and GNAZ-KD mice

	Treatment	
	Control	GNAZ-KD ^1^
Total retinal thickness	231.3 ± 0.9	218.4 ± 1.7***
Retinal sub-layers analysis		
IPL-to-INL		
mean thickness (μm)	97.7 ± 0.7	100.1 ± 1.2
% of total retina	42.5 ± 0.2	45.9 ± 0.8***
OPL-to-ONL		
mean thickness (μm)	75.2 ± 0.7	70.8 ± 1.3**
% of total retina	32.8 ± 0.3	32.4 ± 0.4
IS/OS		
mean thickness (μm)	46.1 ± 0.8	35.5 ± 1.3***
% of total retina	19.4 ± 0.3	16.2 ± 0.5***
RPE		
mean thickness (μm)	12.3 ± 0.1	12.0 ± 0.2
% of total retina	5.3 ± 0.1	5.5 ± 0.1

The right eye of the mice received 1 μL mixture of GNAZ shRNA (20 ng per eye) and jet^OPTIMUS^ transfection reagent by ITV injection, and sham treatment was performed on the left eye. An ophthalmology examination battery was conducted on day 5 after ITV injection. The mice were then euthanized. Their eyeballs were isolated, and homogenates were prepared. GNAZ knockdown in vivo was validated by immunoblotting

Retinal thickness was computed automatically by the software

Data are expressed as mean ± S.E.M. **p < 0.01, ***p < 0.001, indicates statistical difference from the control group. The N values are listed in Additional file 1: Table S1

## Discussion

Blue light has a double-edged effect. During daylight hours, natural exposure to blue light waves from sunshine may boost vigor. Light therapy, which is mostly composed of blue light, is effective in alleviating seasonal depression and improving the relaxation process after stressful events [33, 34]. However, a randomized, double-blind survey cautioned that blue light exposure before bedtime significantly decreases sleep quality [35]. Studies suggest that prolonged exposure to blue light at night not only reduces the secretion of melatonin but also disrupts the circadian rhythm [36]. As the main light-receiving neural tissue, the retina is the primary target of blue light. Previously, acute phototoxicity and histological damage to the retina were demonstrated at illuminances > 500 lx [37, 38]; however, electrophysiological function has not been studied, particularly in general lighting conditions. In this study, mice receiving blue light exposure (80–240 lx, 6 h per day for 3 consecutive days) displayed reduced retinal thickness (with the dominant affected sublayer involving IPL-to-ONL), increased fluorescein leakage (representing the permeable iBRB), attenuated ERG amplitude (especially the b-wave and OPs), and delayed ERG implicit time. This damage was illuminance-dependent, demonstrating the harmful effects of blue light, even without a resulting retinal cell death. Our findings are aligned with the clinical investigation of volunteers with daily terminal video usage cumulative time of over 8 h. This occupational exposure reduces the amplitude of retinal photoreceptors and delays the peak time [39]. Thus, the structural and functional damage of the retina induced by low-illuminance blue light warrants attention.

Layered retinal tissue is processed by a number of types of specialized neurons. The outermost layer consists of photoreceptors and RPE. They are light sensitive. It is well accepted that light, even of ordinary everyday intensity, might deplete their activity; consequently, retinopathy and macular degeneration are displayed over time [40]. Inner retinal neurons are generally considered to be tolerant to light challenges, partially because of their short light-absorbing factors. As mentioned earlier, the dampened ERG b-wave and OPs induced by blue light reflect the obstruction of phototransduction in the OPL, where microenvironment maintenance is achieved by the formation of iBRB. Several studies have shown that iBRB leakage is an early event that implies the development of geographic atrophy and age-related macular degeneration [41]. Persistent downregulation of CLDN5 in mice or primates is sufficient to increase retinal vascular permeability and geographic atrophy, suggesting the contributory role of CLDN5 in developing retinopathy [42]. From our in vitro studies, endothelial CLDN5 was degraded rapidly upon non-cytotoxic illumination (160 lx, 3–6 h), coinciding with the collapse of TJ ultrastructure and reduction of TEER value. In vivo experiments confirmed an increased area of fluorescein leakage and OPs abnormalities; however, no observable histopathology was found. The OPs appearing in the ascending limb of the b-wave are thought to reflect the phototransduction activity of the inner retinal neurons. A reduced amplitude, prolonged implicit time, or both were recorded with circulatory occlusion in the retina or in the early stage of diabetic retinopathy [43, 44]. Again, these data demonstrate that blue light exposure under non-cytotoxic conditions might destroy iBRB, involving the degradation of endothelial CLDN5 protein.

The expression level of CLDN5 could be regulated by transcriptional or post-translational pathways. Previously, we successfully demonstrated CLDN5 as a substrate of ADAM17 [22]. ADAM17 is a member of the adamalysin family that are responsible for the shedding (ectodomain cleavage) of cell membrane proteins. ADAM17 expression has been shown in endothelial cells and different cell types of the CNS [24, 45]. In mammals, the forebrain, where optic vesicle formation is ADAM17-positive, suggests the necessity of ADAM17 for retinal morphogenesis [46]. Although the retinal phenotype of ADAM17^−/−^ mice did not differ from that of wild-type mice, ADAM17 deletion repressed angiogenesis in vitro and disrupted the retinal vasculature in vivo [28, 47]. On the other hand, hyperactivation of ADAM17 may be neurotoxic and implicated in diverse pathologies. For example, increased ADAM17 activity has been observed in diabetic and ischemia–reperfusion-injured retinas. ADAM17 defects effectively prevent vascular leakage and alleviate neurovascular degeneration [48, 49]. In this study, blue light initiates the activation of ADAM17. Additionally, blue-light-mediated CLDN5 degradation can be prevented by pharmacological inhibition or genetic knockdown of ADAM17.

ADAM17 activity is regulated by its modular domains, which correspond to extrinsic stimulation (e.g., thrombin, smoking, and TNFα) and intrinsic signaling factors. Several cytosolic kinases, including protein kinase C, p38-MAPK, and extracellular signal-regulated kinase, are thought to be activators of ADAM17. By T735 phosphorylation, the shedding activity was significantly enhanced. Point mutations at the phosphorylation site did not affect trafficking, but inhibited its activity [25, 26]. In this study, the activation of ADAM17 by blue light was confirmed by increased phosphorylation of T735 in its cytoplasmic domain. Under resting conditions, ADAM17 forms a homodimer on the cell membrane through its cytoplasmic tail, whereas T735 phosphorylation disrupts the dimer into monomers and enables activation of the catalytic domain [50]. Next, the metalloproteinase domain is regularly masked by the endogenous inhibitor, tissue inhibitor of metalloproteinase 3 (TIMP3). Recently, Rhomboid 1 and 2 (iRhoms) were characterized as a complex with ADAM17, thereby facilitating its Golgi-to-membrane trafficking, but silences its activity [51–53]. In this study, for the first time, we found that ADAM17 was sequestered by GNAZ under resting condition.

GNAZ is an inhibitory guanine nucleotide-binding protein (G protein) with unique properties. First, it is immune to pertussis toxin but exhibits long-lasting activity due to the extremely slow hydrolysis of GTP [54, 55]. GNAZ is primarily expressed in neural tissues. Second, GNAZ protein follows a daily rhythmic oscillation with a peak level at night [29]. Thus, GNAZ may be circadian-responsive. Chronic perturbations to the circadian clock have been correlated with several CNS problems (e.g., psychiatric symptoms, sleeplessness, and cognitive impairments) [2]. Several symptoms are implicated in the disturbance of GNAZ and ADAM17. By coupling with Src and the dopamine D2 receptor, GNAZ preferentially stabilizes them in an inactive state [29, 56]. GNAZ-null mice often display neuropsychiatric illnesses relative to dopaminergic hyperactivity [57]. Patients with cognitive deficits show increased plasma ADAM17 activity [58]. These associations support the GNAZ-ADAM17 axis in the maintenance of neural healthy.

Blue light is a potent exogenous regulator of circadian rhythm. Aside of vision formation, the retina coordinates the circadian rhythm. Previously, the rhythmic regulation of GNAZ was shown to be disturbed by hyperglycemia [29], suggesting a putative role of GNAZ in the pathogenesis of diabetic retinopathy. In addition, the attenuation of CLDN5 expression was correlated with defects in circadian-associated proteins. For example, rhythmic expression of CLDN5 was absent in mice with endothelial-specific deletion of *Bmal1* [42, 59]. In addition, *Per2* (a transcriptional repressor of circadian rhythm) mutant mice do not show any metabolic syndromes, but show significant attenuation of CLDN5 expression and an increase in retinal permeability [60]. These findings suggest a relationship between GNAZ, CLDN5, and retinopathy. In the present study, we demonstrated the escape of ADAM17 from the GNAZ upon blue light irradiation. In addition, the hyperactivation of ADAM17, downregulation of CLDN5, and decrease in TEER value were confirmed in vitro using GNAZ-KD endothelial models. Furthermore, we successfully silenced retinal GNAZ in vivo. Weakness of ERG waves and leakage of fluorescein into the fundus were clearly observed. However, we could not conditionally silence the GNAZ of the retinal vessels, in which the majority of GNAZ was found (unpublished data). However, the spatial co-distribution patterns of GNAZ, ADAM17, and CLDN5 in endothelial cells partially confirmed the susceptibility of retinal endothelial cells to blue light as well as the role of retinal endothelial cells in blue-light-mediated retinopathy. In conclusion, for the first time, we demonstrated that blue light exposure may free and activate ADAM17 from GNAZ sequestration, resulting in CLDN5-degraded iBRB leakage and attenuation of phototransduction.

## Supplementary Information

**Additional file 1: Table S1**. Incidence of retinal damages among testing mice. **Figure S1**. Blue light exposure chamber and the illuminances of cell phones. **Figure S2**. Perspectives of the animal treatments. **Figure S3**. Neither the expression level of CLDN5 nor the phosphorylation status of ADAM17 was affected by red light exposure. **Figure S4**. Neither ADAM9 nor ADAM10 participates in blue-light-mediated CLDN5 degradation. **Figure S5**. Histopathology, TUNEL and IHC examinations on retinal tissues.

**Additional file 2:** The source data used throughout the study.

## Data Availability

The datasets used and/or analyzed during the current study are available from the Additional file 2 (source data).

## Abbreviations

LED: Light-emitting diode
iBRB: Inner blood-retinal barrier
RPE: Retinal pigmented epithelium
TJ: Tight junctions
GNAZ: G protein subunit alpha Z
ADAM17: A disintegrin and metalloprotease 17
HREC: Human retinal microvascular endothelial cells
TEER: Transendothelial electrical resistance
FP: Fundus photography
FFA: Fundus fluorescein angiography
SD-OCT: Spectral domain-optical coherence tomography
ERG: Electroretinogram
OPs: Oscillatory potentials
INL: Inner nuclear layer
ONL: Outer nuclear layer
IPL: Inner plexiform layer
OPL: Outer plexiform layer
