# Supplementary material for: Blue light exposure collapses the inner blood-retinal barrier by accelerating endothelial CLDN5 degradation through the disturbance of GNAZ and the activation of ADAM17
Source: Fluids Barriers CNS. 2023 Apr 24;20:31. doi: 10.1186/s12987-023-00430-7 (PMC10124034; doi:10.1186/s12987-023-00430-7)
Supplement: Supplementary file 1 — Additional file 1: Table S1. Incidence of retinal damages among testing mice. Figure S1. Blue light exposure chamber and the illuminances of cell phones. Figure S2. Perspectives of the animal treatments. Figure S3. Neither the expression level of CLDN5 nor the phosphorylation status of ADAM17 was affected by red light exposure. Figure S4. Neither ADAM9 nor ADAM10 participates in blue-light-mediated CLDN5 degradation. Figure S5. Histopathology, TUNEL and IHC examinations on retinal tissues. [file 12987_2023_430_MOESM1_ESM.pdf]

Supplementary Information:

Table S1. Incidence of retinal damages among testing mice.

	Incidence of abnormality					
	Control	Blue light [1]			Red light 240 lux	GNAZ-KD [2]
		80 lux	160 lux	240 lux		
iBRB leakage (FFA)	0/10	4/10	4/10	7/10	0/6	4/6
Retinal architecture destruction (OCT)	0/10	6/10	2/10	6/10	1/6	3/6
Retinal electrophysiology dysfunction (ERG)	0/10	5/10	4/10	8/10	2/6	2/6

Notes:

1. During blue light exposure (80, 160, and 240 lux), C57BL/6 mice were placed in the exposure chamber for 6 h per day (10 AM to 4 PM) for three consecutive days (from day 2 to day 4). An ophthalmology examination battery was performed on days 1 (before exposure) and 5 (post-exposure).
2. The right eye of the mice received 1 μL mixture of GNAZ shRNA (20 ng per eye) and jet^{OPTIMUS} transfection reagent by ITV injection, and sham treatment was performed on the left eye. An ophthalmology examination battery was conducted on day 5 after ITV injection. The mice were then euthanized. Their eyeballs were isolated, and homogenates were prepared. GNAZ knockdown *in vivo* was validated using immunoblotting.

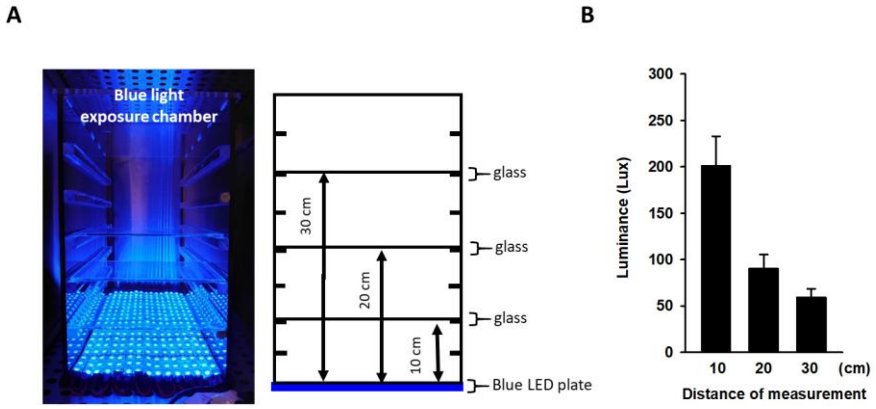

Figure S1. Blue light exposure chamber and the illuminances of cell phones. (A) We have built a blue light chamber for *in vitro* exposures. The blue LEDs were orientated at the bottom of the chamber, and the illuminance was controlled by setting the distance to LED-emitting plate. The illuminance was validated by portable luxmeter before each use. (B) The cell phone illuminance was measured at the distance of 10, 20 and 30 cm from the displayer. Depending on the measures, the intensity of blue light at 80, 160 and 240 lux were used in this study.

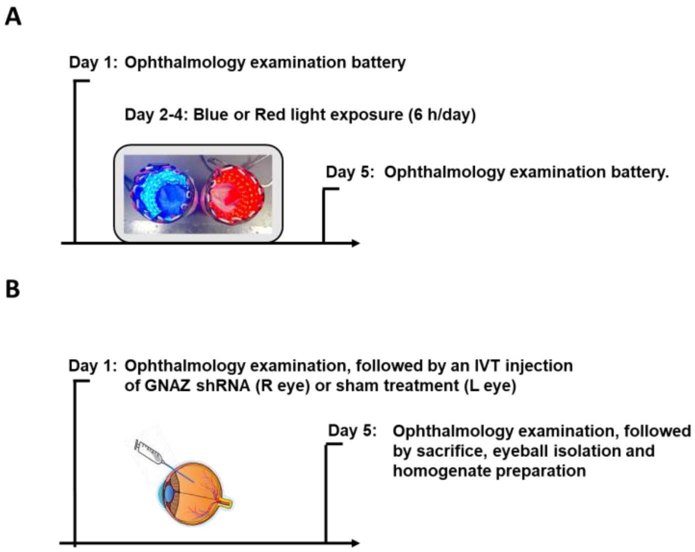

Figure S2. Perspectives of the animal treatments. (A) To study the blue-light-mediated retinopathy, C57BL/6 mice (6-7 weeks old, SPF grade) were randomly divided into a normal control group (n = 10), red light group (n = 6), and blue light group (n = 30). The blue light group was sub-divided into 80, 160, and 240 lux groups. In study day 1, all of the mice were conducted with an ophthalmology examination battery, including fundus photography (FP), fundus fluorescein angiography (FFA), spectral domain-optical optical coherence tomography (SD-OCT), and electroretinogram (ERG). From day 2 to day 4, mice of experimental groups were conditioned in blue light (or red light) exposure chamber for 6 h per day (10 AM to 4 PM). On day 5, the ophthalmology examination battery was conducted again. (B) For GNAZ knockdown study, mice (n = 6) were received a single intravitreal (ITV) injection of 1 μl mixture of GNAZ shRNA (20 ng per eye) and jet^{OPTIMUS} transfection reagent in their right eye. The sham treatment was performed on the left one. Ophthalmology examination battery was performed at 5 days later after ITV injection. The mice were then euthanized. Their eyeballs were isolated, and homogenates were prepared for the validation of GNAZ knockdown.

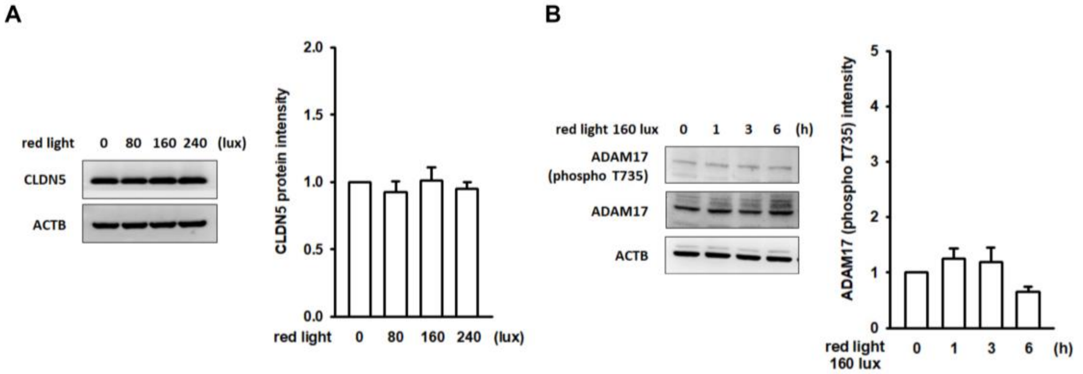

Figure S3. Neither the expression level of CLDN5 nor the phosphorylation status of ADAM17 was affected by red light exposure. Endothelial cell monolayer was exposed to red light, followed by routine immunoblotting. (A) After 6 h, 80-240 lux red light exposure, there was no significant change in the protein level of CLDN5 in treated bEnd.3 cells. (B) The phosphorylation status of ADAM17 was not responded to 160 lux red light stimulation (1-6 h).

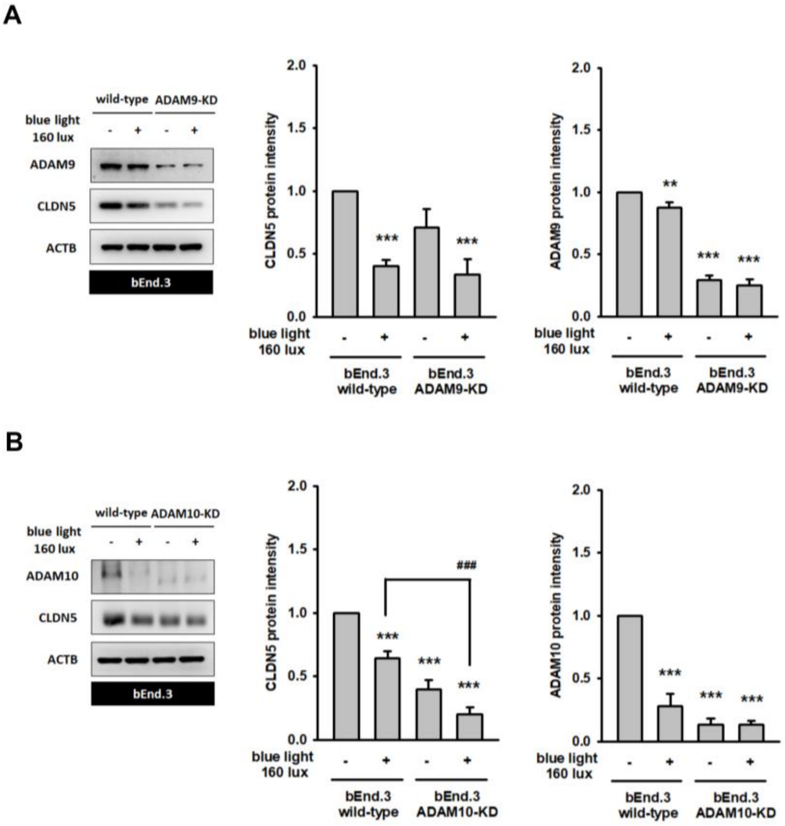

Figure S4. Neither ADAM9 nor ADAM10 participates in blue-light-mediated CLDN5 degradation. ADAM9 and ADAM10 has been reported to express in retinal tissues and are implicated with the retinopathogenesis. The silence of (A) ADAM9 and (B) ADAM10 was achieved by the transfection of shRNA and puromycin selection. Representative images and histograms showed the successful knockdown of ADAM9 and ADAM10, but blue-light-mediated CLDN5 degradation remained unaltered, as compared to that of wild-type. Thus, we thought that ADAM9 and ADAM10 are not responsive to blue light. (*$p < 0.05$, **$p < 0.01$, ***$p < 0.001$, indicates statistically significant difference from the control treatment; $^{###}$ $p < 0.001$ indicates statistical difference from the blue-light-treated group).

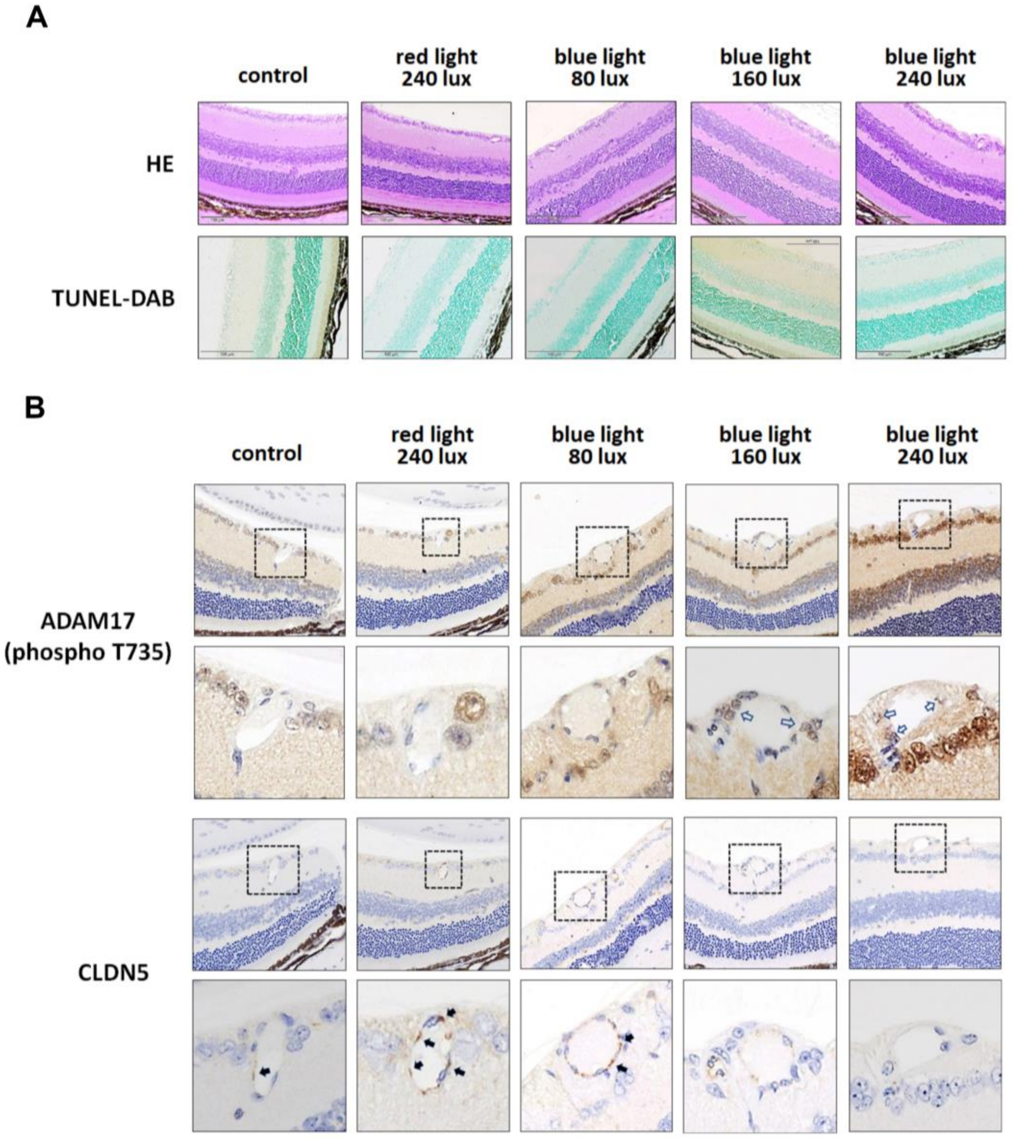

Figure S5. Histopathology, TUNEL and IHC examinations on retinal tissues. Mice treatment was summarized in Fig S2A. At the end of the study, the retinal tissues were isolated for paraffin-embedded tissue block preparation. (A) No histopathological changes or TUNEL-positive lesions were observed in the retinal tissues of any of the test animals. This data supports the *in vivo* findings that "blue light induces iBRB leakage and impairs retinal electrophysiology" was achieved using non-cytotoxic conditions. (B) The performance of CLDN5 and ADAM17 (phospho T735) was detected by IHC staining. The immunoreactivity of CLDN5 expressed specifically in the endothelium of retinal vessels, and it reduced in samples treated with blue light compared to that in the control (black arrow). The phosphorylation status on ADAM17 is inversely correlated with the expression of CLDN5 (white arrow), suggesting the contribution of blue-light-mediated ADAM17 activation in CLDN5 degradation. These results are consistent with our *in vitro* data and partially support the hypothesis that blue-light-mediated iBRB leakage and retinal electrophysiology impairment might be related to ADAM17-driven CLDN5 degradation.
